# Multidimensional sleep health and cognitive function across adulthood

**DOI:** 10.1016/j.sleh.2024.11.005

**Published:** 2025-01-16

**Authors:** Kristin R. Calfee, Soomi Lee, Ross Andel

**Affiliations:** a Department of Human Development and Family Studies, Center for Healthy Aging, Pennsylvania State University, University Park, PA, USA; b Edson College of Nursing and Health Innovation, Arizona State University, Phoenix, AZ, USA; c Memory Clinic, Department of Neurology, Charles University, Second Faculty of Medicine and Motol Hospital, Prague, Czech Republic

**Keywords:** Sleep hygiene, Sleep health composite, Cognition, Executive function, Episodic memory, Aging

## Abstract

**Study objectives:**

Sleep is essential for proper function of the mind and body. Studies report the effect of sleep problems on cognition but focus on only a single or limited number of sleep indicators or on clinical populations (e.g., sleep apnea), and/or provide only cross-sectional results. This study examined cross-sectional and longitudinal associations between multidimensional assessment of sleep health and cognitive function.

**Methods:**

3398 adults (*M*_*age*_ *= 56 years*) provided self-reported sleep and objective cognitive data for the Midlife in the United States study. A subsample of 2119 participants also provided sleep and cognitive data at follow-up approximately 9 years later. A multidimensional, composite measure of sleep health composed of regularity, satisfaction, alertness, efficiency, and duration based on the Ru-SATED model was utilized (higher score = better sleep health) to evaluate self-reported sleep, and cognitive function was assessed using the *Brief Test of Adult Cognition by Telephone*.

**Results:**

Cross-sectionally, better sleep health was associated with better cognition (*B* = 0.121*, SE* = 0.017*, p* < .001). This relationship remained significant even after adjusting for sociodemographic and health covariates (*B* = 0.039*, SE* = 0.014*, p* = .006). Longitudinally, improvement in sleep health from baseline to follow-up was associated with better cognitive performance at follow-up (*B* = 0.031*, SE* = 0.011*, p* = .004); however, this relationship did not remain significant after adjusting for covariates (*B* = 0.015, *p* = .139).

**Conclusion:**

Findings suggest better sleep health measured across multiple domains is associated with higher cognitive function. Future studies may want to examine potential mechanisms by which better sleep health relates to better cognitive function over time, such as reduction in stress or inflammation.

## Introduction

Though the importance of sleep to everyday life is widely recognized, it is often one of the first activities to be sacrificed in favor of other priorities. In 2008, the Center for Disease Control conducted a national survey and found that almost 70% of adults in the United States experience insufficient sleep at least one night per month, and over 11% reported insufficient sleep every night in a 1-month period.^1^ In 2020, a similar report found that 14.5% of adults had trouble falling asleep most or every night over a 1-month period, and 17.8% had trouble staying asleep.^2^ Studies investigating effects of poor sleep demonstrate that insufficient sleep can lead to increased irritability,^3^ difficulties at work,^4^ sensitivity to stress,^5^ and risk of chronic illnesses.^6^

There is also evidence to suggest that lack of optimal sleep can negatively impact cognition.^7–11^ However, there are three important gaps in the literature. *First*, many studies capture only one or a limited number of sleep domains such as duration or quality.^12–15^ There is a growing amount of evidence suggesting that sleep health is not as straightforward as time spent asleep (*duration*) or quality (*satisfaction*) alone.^16–18^ Other aspects of sleep such as taking longer to fall asleep (*latency*), waking up multiple times during sleep (*efficiency*), and irregularity in bedtime and waking schedules (*regularity*) all contribute to one’s sleep health.^19–21^ Thus, a theoretical framework that consider multiple aspects of sleep health is needed.

Buysse’s^17^ recently proposed framework posits several sleep dimensions, Regularity, Satisfaction, Alertness, Timing, Efficiency, and Duration, or Ru-SATED, provide a comprehensive foundation from which to study sleep health as it relates to daily functioning and health outcomes such as physical and psychological wellbeing,^22^ depression,^23^ cardiovascular disease,^24,25^ and physical frailty.^26^ For example, using clinically-derived cutoff points,^24^ Lee et al^22^ showed associations between a poorer score across the Ru-SATED sleep health domains and more perceived stress as well as chronic conditions. The Ru-SATED model reflects our modern knowledge on the importance of positive framing (of “sleep health” rather than sleep disorder) and includes new domains such as regularity that have been shown to have associations with cognition in adults such that less regularity was associated with poorer cognitive function and higher incidence of cognitive impairment.^27–30^

*Second*, while many studies show cross-sectional associations between individual sleep domains and cognition,^11,15,31^ longitudinal associations between multidimensional sleep health and overall cognitive function have been less well explored. Existing longitudinal studies investigating the sleep-cognition relationship typically do so in relatively short time frames (e.g., 1–5 years) and focus on changes in older adults.^32,33^ While cognitive decline typically does not begin until late adulthood, changes in sleep may begin as early as middle adulthood,^22,25^ and sleep may relate differently to cognition in different phases of adulthood,^11^ underscoring the need to include both middle-aged and older adults in this research.

*Third*, previous studies on sleep and cognition largely focused on clinical populations, such as those with sleep-related breathing disorders or cognitive impairment.^34–36^ This limits generalizability, and our ability to understand the link between sleep health and cognition across the middle and older adult population in general.

### Current study

Taking a multidimensional sleep health approach based on the Ru-SATED model,^37^ we examined whether sleep health is associated with cognitive function in a national sample of adults in the United States. Cross-sectionally, we hypothesized that better sleep health at baseline would be associated with better cognitive function at baseline. Longitudinally, we hypothesized that an improvement in sleep health over time would be associated with better cognitive function at follow-up after controlling for baseline cognitive function. In examining these associations, we considered an extensive list of covariates known to be related to sleep and cognition (e.g., education, depression^38,39^) to reduce the influence of potential confounding factors.

## Methods

### Participants and procedures

Data from two timepoints (2004–2007 and 2013–2016) of the Midlife in the United States (MIDUS) study, including the self-administered questionnaire (SAQ), cognitive project, and Milwaukee projects were used. Participants in the MIDUS study were sampled using random-digit-dialing to create a nationally representative sample of adults from various areas of the United States.^40^ The Milwaukee projects oversampled Black adults in the Milwaukee metropolitan area to increase racial/ethnic diversity of the overall MIDUS sample. Efforts were made to procure similar distributions of other demographic characteristics between the samples; however, the Milwaukee sample (n = 592) had proportionally more female participants (χ^2^ = 17.9, *p* < .001), were slightly younger on average (*t*_*(5552)*_ = 7.04, *p* < .001), and had lower educational attainment on average (*t*_*(773.1)*_ = 15.4, *p* = < .001) compared to the core sample (n = 4963). Individuals were eligible for participation in the MIDUS study if they were between 25 and 74 years of age during the first wave of data collection (1995–1997), spoke English, were not institutionalized, and lived in the coterminous United States.^40^ Individuals were not excluded for any health or neurological conditions. Cognitive data were collected in the second (2004–2007) and third (2013–2016) waves, which served as Time 1 (T1) and Time 2 (T2), respectively, in the current analysis.

Participants completed all survey questions via phone and were contacted at a later time to participate in the cognitive project, which included administration of the *Brief Test of Adult Cognition by Telephone (BTACT)*. Those who completed the SAQ during T1 were contacted again approximately 9 years later to participate in T2. Further details regarding MIDUS study design and procedures can be found in previous literature.^40^ The MIDUS studies were approved by the University of Wisconsin-Madison Institutional Review Board (IRB). Written informed consent was received for all MIDUS participants. The current study was exempt from an IRB review due to the use of publicly available, deidentifiable data.

**Fig. 1.**
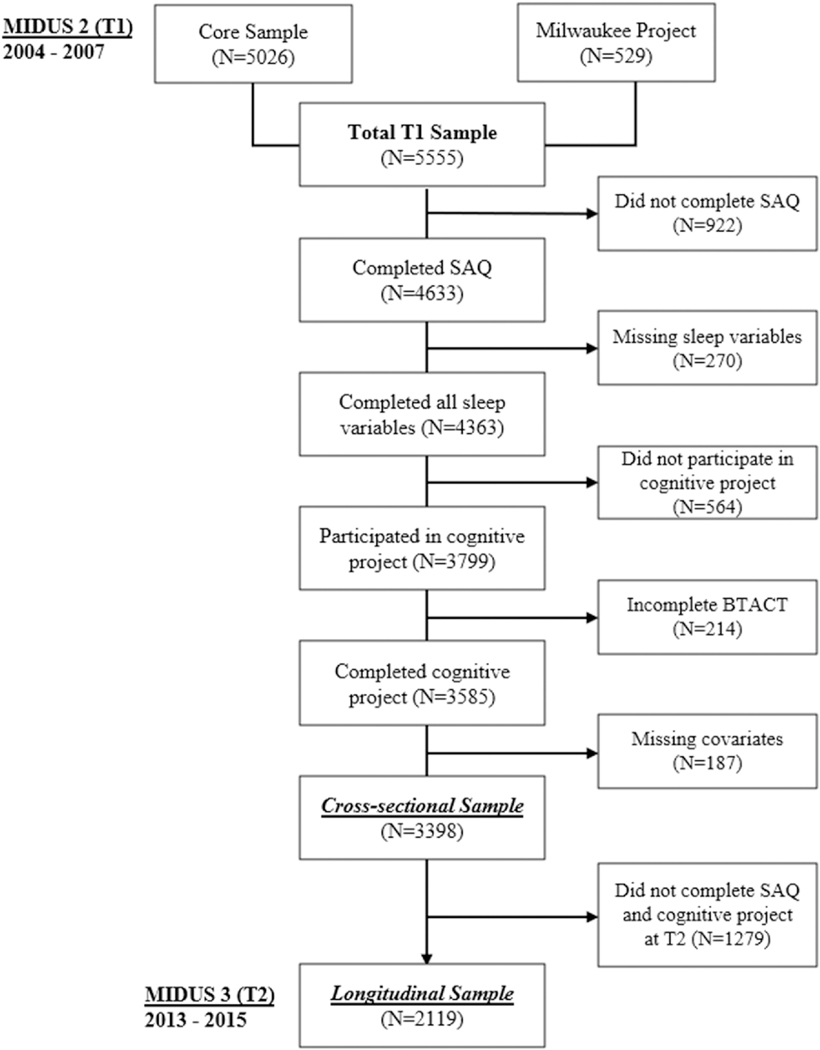
Flowchart of participant exclusion. BTACT, the Brief Test of Adult Cognition by Telephone; MIDUS, Midlife in the United States study; SAQ, self-administered questionnaire; T1, Timepoint 1; T2, Timepoint 2

Data from 5555 individuals were collected during T1. Of the 4633 participants who completed the SAQ, 4363 answered all sleep questions needed to calculate sleep health composite scores, and 3585 participants also completed the cognitive project. A total of 3398 participants had complete data for all sociodemographic and health status covariates and were used in the cross-sectional analysis at T1. A total of 2119 participants completed all sleep variables, cognitive variables, and covariates at both timepoints and were included for longitudinal analysis (62.4% retention rate). Fig. 1 shows a comprehensive flowchart of participant exclusion.

### Measures

*Sleep Health Composite Score* was calculated using the Ru-SATED model sleep dimensions.^37^ This model combines six dimensions of self-reported sleep measures: regularity, satisfaction, alertness, timing, efficiency, and duration. Timing, however, was not captured through the MIDUS survey and therefore was not included in the composite score for this study. Remaining sleep domains were collected from participants’ self-rated sleep from the past month. Each domain was dichotomized (1 = *optimal*, 0 = *suboptimal*) with established cutoff points (Table 1) which were validated in previous literature.^22–26,37,41^ Regularity was determined by the difference between sleep duration on workdays and nonworkdays. An absolute difference of less than 60 minutes was scored as *regular (optimal)*. Satisfaction consisted of four items: trouble falling asleep, nocturnal awakenings (i.e., waking up during the night and difficulty going back to sleep), early awakenings (i.e., waking up too early in the morning and unable to get back to sleep), and feeling unrested during the day. To calculate the composite score, each satisfaction item was dichotomized (0 = *sometimes*, *often*, or *almost always;* 1 = *never* or *rarely*), and a sum of 1 or less was considered *satisfactory (optimal).* Alertness was measured by frequency of naps lasting more than 5 minutes. Two or fewer naps per week was considered *sufficient alertness (optimal)*. Efficiency was measured as minutes to fall asleep. Thirty minutes or less was considered *efficient (optimal)*. Duration was measured by the number of hours usually slept at night on workdays. Between 6 and 8 hours of sleep was considered age-appropriate sleep duration *(optimal)*. A sum of scores across the five domains was used as the composite sleep health score (Range = 0–5), with a score of 0 indicating suboptimal sleep in all five domains, and a score of 5 indicating the most optimal sleep health.

BTACT is a well-validated measure of cognitive performance in adults and covers a variety of cognitive domains shown to be important for assessment of cognitive aging including episodic memory (EM) and executive function (EF).^42^ The BTACT includes seven subtests: word list recall, digit span forward, digit span backwards, category fluency, stop and go task (SGTS), number series, and 30-second counting task (30-SACT). Raw scores for each subtest were transformed to z-scores and averaged to create a composite score for overall cognitive performance. Z-scores for backwards digit span, number series, category fluency, SGTS, and 30-SACT were averaged to create the EF score. Immediate word list recall and delayed word list recall subtest z-scores were averaged to create the EM score.^43^ Per MIDUS protocol, z-scores at T2 were calculated using T1 means and standard deviations.

### Covariates

Fully adjusted models controlled for sociodemographic and health covariates found in previous literature^38,39,44^ to be associated with sleep and cognitive function including age (in years), sex (1 = female, 0 = male), race/ethnicity (1 = non-Hispanic White, 0 = all other races/ethnicities), education level (scale of 12 = PhD, MD, or other professional degree to 1 = no school or some grade school; more details outlined in Table 2), depression symptoms, body mass index (BMI) (kg/m^2^), smoking (1 = current smoker, 0 = never or former smoker), consumption of at least one alcoholic beverage in the past month (1 = drank alcohol, 0 = did not drink), and employment status (1 = regularly working for pay, 0 = not working for pay). Depression symptoms were measured using the World Mental Health Organization’s Composite International Diagnostic Interview Short Form.^45^ One question regarding sleep (initial insomnia) was excluded from the depression score.^25^ The remaining six depression symptoms were added (1 = symptom present, 0 = symptom not present) for a composite score ranging from 0–6, where 0 represents no depression symptoms. Time-varying covariates in longitudinal analyses were measured at T2.

### Statistical analysis

Descriptive statistics and correlations were used to examine sample characteristics and univariate associations between main variables. To test our hypotheses, we used multivariable linear regression models (procedure GLM) in SAS (SAS Institute, Cary, NC, version 9.4). In cross-sectional analyses, we tested the association between sleep health composite and T1 BTACT score. In longitudinal analyses, we tested two separate models: the first regressing T2 BTACT score onto T1 sleep health composite score, and the second regressing T2 BTACT score onto change in sleep health composite score from T1 to T2 while controlling for T1 sleep health composite. Fully adjusted models for all cross-sectional and longitudinal analyses included age, sex, race/ethnicity, education level, depression symptoms, BMI, current smoking status, alcohol consumption, and employment status as covariates. Longitudinal models used T2 values for time-varying covariates and were also controlled for T1 BTACT score.

### Supplemental analysis

First, we further investigated the relationship between sleep and cognition by analyzing each individual sleep dimension (regularity, satisfaction, alertness, efficiency, and duration) in relation to BTACT at T1 and T2. We used the raw, continuous variables of individual sleep dimensions (i.e., before dichotomization) to provide a more detailed representation of the relationships. Sleep variables at T1 were used for cross-sectional models, and change scores for each sleep variable were used in longitudinal models. Second, we examined the cognitive subscales of the BTACT, EF and EM, in relation to sleep health composite score in separate models. Third, we investigated the potential role of age in the relationship between sleep health composite and BTACT score. We created a categorical variable by grouping age into categories using the T1 sample median ± 1 SD: ≤44 years, 45–67 years (reference), ≥68 years, which was then used as a moderator. To further examine the potential role of age, this age grouping was also used to stratify the sample to evaluate the sleep-cognition relationship within each age group.

## Results

### Descriptive statistics

Sample characteristics of the final analytic samples (*N*_*cross-sectional*_ = 3398 and *N*_*longitudinal*_ = 2119) and descriptive statistics are displayed in Table 2. At T1, participants were between 32 to 84 years of age (*M* = 55.6, *SD* = 12.1), 44.2% were male, 85.5% identified as non-Hispanic White, and the average level of education was 3 or more years of college with no degree. Average sleep health composite score was 3.35 (*SD* = 1.05), meaning that participants were sufficient in approximately three out of five sleep domains on average. Sample characteristics for participants at T1 and T2 were similar except a higher proportion of participants at T2 not working for pay (47% to 57%). Participants who were included in cross-sectional analyses only (n = 1279) did not significantly differ from the longitudinal sample (n = 2119) in age at T1, sex, race, or level of education.

Table 3 shows correlations among main variables. T1 Sleep health composite score was moderately correlated with T2 sleep health composite score (*r* = 0.45, *p* < .001). Sleep health composite score and individual sleep dimensions were moderately correlated (0.39≤ *r* ≤0.57), indicating that the sleep dimensions were related, but each provided unique information about overall sleep health. T1 BTACT score was strongly correlated with T2 BTACT score (*r* = 0.78*, p* < .001). BTACT score was also highly correlated with EF and EM subdomains at T1 (*r*_*EF*_ = 0.94, *p* < .001; *r*_*EM*_ = 0.64, *p* < .001) and T2 (*r*_*EF*_ = 0.90, *p* < .001; *r*_*EM*_ = 0.62, *p* < .001). Tests of linearity between the main variables, normality of residuals, homoscedasticity, and independence were conducted and demonstrated that the assumptions for use of linear regression were not violated. Model fit was adequate for fully adjusted cross-sectional (*RMSE* = 0.80) and longitudinal models using T1 sleep health composite (*RMSE* = 0.39) change in sleep health score (*RMSE* = 0.39).

### Cross-sectional association between sleep health and cognitive function

Table 4 displays results for unadjusted and fully adjusted models testing cross-sectional associations between sleep health and BTACT score. The unadjusted model revealed that a higher sleep health composite score was concurrently associated with a higher BTACT score (*B* = 0.121*, SE* = 0.017*, p* < .001). This association weakened after adjusting for the significant associations of being female, non-Hispanic White, younger, having higher education, drinking alcohol in the past month, and working for pay, but remained significant (*B* = 0.039*, SE* = 0.014*, p* = .006) such that each additional optimal sleep characteristic was associated with a 0.039 standard deviation higher BTACT score.

### Longitudinal association between sleep health and cognitive function

Table 5 displays results from models testing longitudinal associations between change in sleep health composite score and subsequent BTACT scores. When controlling for sleep health composite score and BTACT score at T1, an increase in sleep health composite score (improvement in sleep health) from T1 to T2 had a significant association with higher BTACT scores at T2 (*B* = 0.031*, SE* = 0.011*, p* = .004); however, this association did not remain significant (*B* = 0.015, *SE* = 0.020, *p* = .139) after adjusting for sociodemographic and health covariates. Furthermore, sleep health at T1 was not significantly related to T2 BTACT score when modeled without change in sleep health in the unadjusted (*B* = 0.006*, SE* = 0.009*, p* = .481) nor fully adjusted models (*B* = 0.005*, SE* = 0.009*, p* = .575).

### Supplemental results

In the first set of supplemental analyses, we analyzed individual sleep dimensions and their respective associations with cognitive function (Table A.1). Cross-sectionally, only sleep efficiency had significant associations with T1 BTACT score in the fully adjusted model such that more efficient sleep was associated with a higher BTACT score. Longitudinally, none of the sleep dimensions were significant correlates of subsequent BTACT score after adjusting for covariates.

In the second set of supplemental analyses, we used cognition subdomain scores of the BTACT as outcomes (Table A.2). Results from the analyses using EF subdomain scores were generally consistent with those for overall cognitive function. A higher sleep health composite score was cross-sectionally associated with a higher EF score in both the unadjusted and adjusted models, but there were no longitudinal associations in the unadjusted nor fully adjusted models. EM was associated with sleep health composite in the unadjusted cross-sectional model, but was neither cross-sectionally nor longitudinally associated with sleep health in fully adjusted models.

In the third set of supplemental analyses, we explored a possible role of age in the sleep health and cognition relationship (Table A.3). Neither the cross-sectional nor longitudinal models showed significant interaction effects between sleep health composite and age group in relation to BTACT score, nor were there significant associations between change in sleep health and T2 cognition when stratified by age group.

## Discussion

The current study investigated the relationship between sleep health and cognitive function in a sample of U.S. adults. The use of a sleep health composite score based on the Ru-SATED (regularity, satisfaction, alertness, efficiency, and duration) model,^37^ and a nonclinical sample of U.S. middle-age and older adults are unique strengths of this study. Moreover, we examined both cross-sectional and longitudinal associations between sleep health and cognition. Results from the cross-sectional analyses supported our hypothesis that better sleep health would be associated with a higher BTACT score even when controlling for sociodemographic and health covariates. Longitudinally, the hypothesis that an improvement in sleep health from T1 to T2 would be associated with a higher BTACT score at T2 was no longer supported after adjusting for covariates. Overall, these findings demonstrate a robust association between sleep health and concurrent cognitive function. Long-term changes in sleep health may function as an indicator for future cognitive function, albeit this association may be dependent on sociodemographic and health characteristics.

Although many cross-sectional studies report associations between sleep and cognition,^8–10^ our results still advance the literature in several ways. This study is unique in its utilization of a sleep health composite that combines multiple domains of sleep based on the Ru-SATED model, whereas many studies only consider sleep duration and/or quality. Our findings show that the sleep health composite explained unique variance in cognitive function beyond individual sleep domains. Notably, sleep duration, which is one of the most widely studied domains of sleep, was not associated with overall cognitive function. Instead, sleep efficiency, measured by optimal sleep onset latency (≤30 minutes) was independently associated with higher cognitive function in supplemental analysis. These results indicate that sleep efficiency may be the driving force behind the observed cross-sectional associations between sleep health and cognition and an important component of overall sleep health. Sleep onset latency longer than 30 minutes is often used as a sign for sleep-onset insomnia in clinical practice,^46^ and is found to impair cognition in a laboratory-based study.^47^

The inclusion of regularity is a feature of the Ru-SATED model that distinguishes it from other commonly used sleep health scales such as the PSQI global sleep score.^48^ Although some studies have found sleep regularity to be associated with better cognitive function, we did not find significant associations between sleep regularity and BTACT score in this sample of adults, which more closely resembles findings in older adult samples. Regardless of these null findings, in looking at individual sleep domains such as quality or duration, we may be neglecting key components of sleep and underestimating the importance of sleep health for cognitive function. In a randomized crossover trial study,^30^ regular *adequate* sleep over a period of 6 weeks resulted in better cognitive performance, whereas regular *inadequate* sleep did not. These findings highlight the multidimensionality of sleep health and demonstrate that examining the various domains of sleep in isolation may be overlooking important interactions between them and potential associations of these interactions with cognition.

Though longitudinal increase in sleep health seemed associated with higher cognitive function at follow-up, this association disappeared after controlling for well-known sociodemographic and health risk factors. This may relate to “social determinants of health,” which suggests that those with fewer social resources are likely to experience worse health.^49^ Thus, sociodemographic characteristics may explain more variance in cognitive function compared to sleep health. It is also possible that mechanisms not explored in this study might have played a role. For example, better sleep health may decrease perceived stress,^22^ and decreased stress may be associated with improvement in cognitive function.^50^ Change in sleep may also accompany change in lifestyle behaviors (e.g., engagement in physical or cognitive activities), which likely occurs over the course of aging and is important for subsequent cognitive function. Similarly, although our cross-sectional results associating better sleep health with higher BTACT score reached statistical significance in the fully adjusted model, the small effect size after adjusting for sociodemographic and health covariates may limit clinical significance of the findings and may indicate that these “social determinates of health” play a role both cross-sectionally and longitudinally. Future studies can extend the current analyses by testing whether sleep health has indirect associations with cognitive function through stress and other behavioral or neurological mechanisms.

Additionally, T1 sleep health was not significantly related to T2 cognition in our sample. This, combined with our cross-sectional results, suggests that sleep health may have a more proximal influence on cognitive function. However, the time gap between data collection timepoints limits our ability to determine sleep patterns over time and does not account for fluctuations in sleep health between timepoints. Furthermore, with only two timepoints, the longitudinal data might have been affected by the regression to the mean phenomenon. Therefore, longitudinal examination of these associations with both a greater follow-up time and more frequent measurements is needed to provide support for or to refute these initial findings.

This study adds empirical evidence on the relationship between sleep health and cognition in a large community-dwelling sample of U.S. adults. Whereas much of the previous literature on this relationship has been specific to clinical populations such as those with sleep apnea or cognitive impairment,^34–36^ this study is not limited to a sample with any common clinical feature which allows for greater generalizability. We also considered an extensive list of sociodemographic and health covariates that may relate to both sleep health and cognition. The cross-sectional association between sleep health and BTACT remained significant even after controlling for these covariates, which suggests that sleep health is an independent factor for concurrent cognitive function beyond the associations of known risk factors (older age, male sex, lower education, etc.).^38,39,44^ Consistent findings across age groups found in our supplemental analyses may suggest that sleep health is important to cognition regardless of age group, and that sleep health should be promoted in the general public from early adulthood.

### Limitations and future directions

This study is not without limitations. First, the sleep timing component of the Ru-SATED model was not captured with the self-reported sleep measures in the MIDUS data and was therefore not included in the sleep health composite score. Future research that includes sleep timing in the calculation of sleep health composite score can examine sleep health even more comprehensively. Additionally, only self-reported sleep measures were used due to both sleep actigraphy and cognitive data being available at both timepoints for only a limited number of participants (n = 73). Future research could use the combination of actigraphy and self-report to best capture each sleep dimension (see Brindle et al^24^ and Lee et al^25^ for examples) and examine their possible relationship with cognitive function.

Moreover, the MIDUS sample used in this analysis was, on average, relatively privileged in terms of race, education, and health. Previous literature and the findings from this study suggest that sociodemographic characteristics have a significant impact on both sleep and cognitive function. Replication of this study using a sample of adults with greater sociodemographic diversity may better represent the general population and can investigate how the relationship between sleep and cognition may differ for different populations. There are also possible limitations of administering cognitive testing over the phone (e.g., phone connection issues, call quality, and hearing difficulties); however, previous assessment of this potential issue with the MIDUS sample demonstrated that this method of administration of the BTACT did not significantly differ from in-person cognitive testing.^43^ We also controlled for employment status, but our variable did not distinguish between retirees vs. those not working for other reasons, which may potentially have affected results. Finally, the 9-year follow-up interval allowed for a uniquely long follow-up, but may underestimate heterogeneity of change occurring between timepoints. Future research should also investigate a long follow-up but include more frequent assessments within that time span.

## Conclusion

This study adds to the sleep and cognitive literature by demonstrating that better sleep health assessed by multiple dimensions is a robust correlate of higher concurrent cognitive function in adulthood. Moreover, long-term improvement in sleep health may function as an indicator for improvement in cognitive function over that time period, albeit this association may be dependent on well-known sociodemographic characteristics and health risks. Sleep health is a modifiable factor that influences cognitive aging, and our findings suggest that good sleep health should be considered as one of the key lifestyle behaviors that may promote cognitive health.

## Supplementary Material

Supplemental Tables

## Figures and Tables

**Table 1:** Sleep health dimensions and cut points used to construct sleep health composite score

Sleep health composite			

Dimension	Variable	Assessment/Item	Cut point

Regularity	Sleep debt	Difference between workday sleep duration and nonworkday sleep duration	1: Absolute value ≤60 min 0: Absolute value > 60 min
Satisfaction	Trouble falling asleep Nocturnal awakenings Early awakenings Unrested upon waking	Have trouble falling asleep. Wake up during the night and have difficulty going back to sleep. Wake up too early in the morning and be unable to get back to sleep. Feel unrested during the day, no matter how many hours of sleep you had.	1: Rarely or Never 0: Sometimes, Often, or Almost Always 1: Score of 1 for all 4 items 0: Score of 0 on at least 1 of 4 items
Alertness	Nap frequency	During a usual week, how many times do you nap for 5 min or more?	1: ≤2 naps per week 0: > 2 naps per week

Timing	Not captured through survey*		

Efficiency	Sleep onset latency	How long does it usually take you to fall asleep at bedtime?	1: ≤30 min 0: > 30 min
Duration	Workday sleep duration	How much sleep do you usually get at night (or in your main sleep period) on weekdays or workdays?	1: ≥6 and ≤8 h 0: < 6 or > 8 h

Cut points were determined using empirical evidence from previous research.^25^ The sum across the binary variables was calculated, such that higher scores represented better sleep health.

* indicates that the correlation coefficient is significant at p < .05, or that the p value for the coefficient is greater than .05

**Table 2:** Descriptive statistics of the analytical sample (N_T1_ = 3398, N_T2_ = 2119)

	T1 mean (SD) or n (%)	T2 mean (SD) or n (%)

Primary measures		
BTACT z-score	0.046 (0.98)	0.017 (0.68)
Sleep health composite score	3.35 (1.05)	3.30 (1.05)
0	16 (0.5%)	8 (0.38%)
1	152 (4.5%)	101 (4.8%)
2	500 (14.7%)	338 (16.0%)
3	1125 (33.1%)	716 (33.8%)
4	1164 (34.3%)	710 (33.5%)
5	441 (13.0%)	246 (11.6%)

Covariates		
Male	1502 (44.2%)	913 (43.1%)
Non-Hispanic white	2906 (85.5%)	1826 (86.2%)
Age (in y)	55.6 (12.11)	63.6 (10.70)
Education^a^	7.29 (2.51)	7.64 (2.49)
BMI (kg/m^2^)	28.17 (5.95)	28.5 (6.30)
Depression symptoms	0.53 (1.51)	0.9 (2.64)
Drank alcohol in past month	2044 (60.0%)	1307 (61.7%)
Current smoker	504 (14.8%)	208 (9.8%)
Not working for pay	1588 (46.7%)	1204 (56.8%)

Abbreviations: BMI, body mass index; BTACT, the Brief Test of Adult Cognition by Telephone; T1, Timepoint 1; T2, Timepoint 2.

Higher sleep health composite score indicates better sleep health.

a Education on scale of 0–12 and ranged from no school or some grade school = 0 to postgraduate or professional degree (e.g., PhD, MD, etc.) = 12; the mean level of 7.29 and 7.64 respectively are equivalent to completing 3 years of college but no degree earned.

**Table 3:** Correlation of sleep and cognitive variables at T1 and T2 (N_T1_ = 3398, N_T2_ = 2119)

Variables	Sleep HCS	Regularity	Satisfaction	Alertness	Efficiency	Duration	BTACT
Sleep HCS	.45*	.39*	.57*	.47*	.53*	.53*	.12*
Regularity	.40*	.27*	.05*	−.03	.03	.10*	−.10*
Satisfaction	.57*	.03	.39*	.05*	.21*	.09*	−.01
Alertness	.50*	−.02	.08*	.37*	.02	.02	.18*
Efficiency	.54*	.03	.21*	.02	.42*	.15*	.11*
Duration	.50*	.02	.08*	.06*	.16*	.26*	.07*
BTACT	.12*	−.07*	.00	.15*	.12*	.10*	.78*

Abbreviations: BTACT, the Brief Test of Adult Cognition by Telephone; HCS, Health Composite Score.

Lower triangle comprises of correlations for timepoint 1 (T1); upper triangle comprises of correlations for timepoint 2 (T2). Diagonal comprises of correlation between T1 and T2. Higher values for sleep variables indicate better sleep. Higher values for cognitive variables indicate better cognitive performance.

* *p* < .05.

**Table 4:** General linear modeling of BTACT score and sleep health score at T1 (N = 3398)

	*B*	95% CI		*p*-value

Main effect				
Sleep health composite score (unadjusted)	0.121	0.089	0.155	**< .001**
Sleep health composite score (adjusted)	0.039	0.011	0.067	**.006**
Covariates				
Male	−0.077	−0.132	−0.022	**.006**
Non-Hispanic white	0.545	0.466	0.623	**< .001**
Age	−0.319	−0.351	−0.287	**< .001**
Education^a^	0.133	0.122	0.144	**< .001**
Depression symptoms	−0.010	−0.038	0.018	.498
BMI (kg/m^2^)	−0.016	−0.045	0.014	.296
Current smoker	−0.054	−0.132	0.024	.176
Drank alcohol in past month	0.090	0.033	0.147	**.002**
Not currently working for pay	−0.062	−0.123	−0.001	**.047**

Abbreviations: BMI, body mass index; BTACT, the Brief Test of Adult Cognition by Telephone; T1, Timepoint 1.

Significance level set to α = .05.

Bolding indicates that the *p* value is above .05

a Education on scale of 0–12 where no school or some grade school = 0 to post-graduate or professional degree (e.g., PhD, MD, etc.) = 12.

**Table 5:** General linear modeling of longitudinal relationship between T2 BTACT score and T1 sleep health composite score, and T2 BTACT score and change in sleep health composite score

Longitudinal (N = 2119)				

	*B*	95% CI		*p*-value

Main effect				
T1 sleep health composite score (unadjusted)	0.006	−0.011	0.024	.481
T1 sleep health composite score (adjusted)	0.005	−0.012	0.022	.575
Covariates				
T1 BTACT score	0.462	0.440	0.483	**< .001**
Male	−0.028	−0.063	0.007	.115
Non-Hispanic white	0.097	0.051	0.144	**< .001**
Age	−0.014	−0.016	−0.012	**< .001**
Education^a^	0.029	0.022	0.037	**< .001**
Depression symptoms	−0.007	−0.014	−0.001	**.032**
BMI (kg/m^2^)	0.001	−0.003	0.002	.337
Current smoker	−0.055	−0.114	0.004	.067
Drank alcohol in past month	0.016	−0.020	0.052	.377
Not currently working for pay	−0.039	−0.079	0.001	.055

	*B*	95% CI		*p*-value

Main effect				
Change in sleep health comp. score (unadjusted)	0.031	0.010	0.053	**.004**
Change in sleep health comp. score (adjusted)	0.015	−0.005	0.035	.139
Covariates				
T1 sleep health composite score	0.011	−0.009	0.030	.297
T1 BTACT score	0.460	0.436	0.479	**< .001**
Male	−0.028	−0.063	0.007	.116
Non-Hispanic white	0.113	0.061	0.166	**< .001**
Age	−0.014	−0.016	−0.012	**< .001**
Education^a^	0.028	0.020	0.035	**< .001**
Depression symptoms	−0.005	−0.012	0.001	.123
BMI (kg/m^2^)	0.001	−0.002	0.004	.382
Current smoker	−0.053	−0.111	0.006	.076
Drank alcohol in past month	0.012	−0.023	0.049	.471
Not currently working for pay	−0.029	−0.069	0.012	.163

Abbreviations: BMI, body mass index; BTACT, the Brief Test of Adult Cognition by Telephone; T1, Timepoint 1; T2, Timepoint 2.

Significance level set to α = .05.

Bolding indicates that the *p* value is above .05

a Education on scale of 0–12 where no school or some grade school = 0 to postgraduate or professional degree (e.g., PhD, MD, etc.) = 12.

## Data Availability

The data underlying this article are available in Inter-university Consortium for Political and Social Research (ICPSR), at https://dx.doi.org/10.3886/ICPSR04652.v8 (MIDUS 2 Core Sample), https://dx.doi.org/10.3886/ICPSR22840.v5 (MIDUS 2 Milwaukee Project), https://dx.doi.org/10.3886/ICPSR25281.v6 (MIDUS 2 Cognitive Project), https://dx.doi.org/10.3886/ICPSR36346.v7 (MIDUS 3 Core Sample), https://dx.doi.org/10.3886/ICPSR37120.v2 (MIDUS 3 Milwaukee Project), https://dx.doi.org/ICPSR37095.v2 (MIDUS 3 Cognitive Project).

## Appendix A. Supporting information

Supplementary data associated with this article can be found in the online version at doi:10.1016/j.sleh.2024.11.005.
