# Supplementary material for: Multidimensional sleep health and cognitive function across adulthood
Source: Sleep Health. Author manuscript; Available in PMC 2025 Dec 23. (PMC12724623; doi:10.1016/j.sleh.2024.11.005)
Supplement: Supplemental Tables [file NIHMS2125716-supplement-Supplemental_Tables.pdf]

Supplemental Material

Table A.1 General linear modeling of individual sleep domains in relation to cognition (BTACT)

Cross-sectional at T1 (N=3398)				
	B	***95% CI***		***p-value***
Sleep health composite score	0.039	0.011	0.067	**.006**
Regularity	-0.021	-0.048	0.007	.140
Satisfaction	0.018	-0.002	0.037	.072
Alertness	0.006	-0.006	0.017	.337
Efficiency	0.114	0.058	0.170	**<.001**
Duration	-0.020	-0.044	0.003	.091

Longitudinal (N=2119)				
	B	***95% CI***		***p-value***
Change in sleep health composite score	0.015	-0.005	0.035	.139
Regularity	-0.002	-0.022	0.017	.810
Satisfaction	0.009	-0.005	0.023	.227
Alertness	0.007	-0.001	0.014	.107
Efficiency	0.035	-0.003	0.072	.068
Duration	-0.009	-0.025	0.008	.296

Note: significance level $\alpha = .05$. Higher sleep health composite score indicates better sleep. Each dimension was entered in a separate model. Fully adjusted models control for age, sex, race/ethnicity, education, depression symptoms, body mass index, smoking status, alcohol use, and employment status. Sleep health composite parameters from main analysis included for comparison. T1 = Timepoint 1; BTACT = the Brief Test of Adult Cognition by Telephone.

Table A.2 General linear modeling of sleep health score in relation to cognitive subdomains from BTACT

Cross-sectional at T1 (N=3398)				
	B	***95% CI***		***p-value***
**Executive function**				
Sleep health composite score (unadjusted)	0.117	0.085	0.149	**<.001**
Sleep health composite score (adjusted)	0.030	0.003	0.057	**.027**
**Episodic memory**				
Sleep health composite score (unadjusted)	0.054	0.022	0.087	**.001**
Sleep health composite score (adjusted)	0.029	-0.002	0.060	.065

Longitudinal (N=2119)				
	B	***95% CI***		***p-value***
**Executive function**				
T1 sleep health composite (unadjusted)	0.007	-0.012	0.027	.468
T1 sleep health composite (adjusted)	0.004	-0.015	0.023	.658
Change in sleep health composite (unadjusted)	0.013	-0.010	0.036	.274
Change in sleep health composite (adjusted)	-0.002	-0.024	0.020	.836
**Episodic memory**				
T1 sleep health composite (unadjusted)	0.009	-0.027	0.044	.622
T1 sleep health composite (adjusted)	0.008	-0.026	0.043	.632
Change in sleep health composite (unadjusted)	0.050	0.008	0.091	**.018**
Change in sleep health composite (adjusted)	0.033	-0.007	0.072	.107

Note: significance level α = .05. Fully adjusted models control for age, sex, race/ethnicity, education, depression symptoms, body mass index, smoking status, alcohol use, and employment status. T1 = Timepoint 1; T2 = Timepoint 2; BTACT = the Brief Test of Adult Cognition by Telephone.

Table A.3 Associations between sleep health and cognition (BTACT) within each age group and as moderated by age group

Cross-sectional at T1 (N=3398)				
	B	***95% CI***		***p-value***
**Models stratified by age group**				
Sleep health composite				
Age ≤ 43 yrs (n=629)	0.039	-0.026	0.104	.239
Age 44 – 66 yrs (n=2074)	0.029	-0.005	0.063	.092
Age ≥ 67 yrs (n=695)	0.050	-0.007	0.107	.083
**A model with age group-by-sleep interaction**				
Sleep health composite	0.027	-0.006	0.061	.111
Age ≤ 43 yrs	0.202	-0.033	0.437	.093
Age ≥ 67 yrs	-0.730	-0.976	-0.484	**<.001**
Sleep health composite*age ≤ 43	0.031	-0.036	0.098	.363
Sleep health composite*age ≥ 67	0.044	-0.026	0.114	.220
**Longitudinal (N=2119)**				
	B	***95% CI***		***p-value***
**Models stratified by age group**				
Sleep health composite				
T1 Age ≤ 43 yrs (n=400)	0.019	-0.022	0.059	.366
T1 Age 44 – 66 yrs (n=1400)	0.015	-0.007	0.038	.175
T1 Age ≥ 67 yrs (n=287)	0.008	-0.046	0.062	.777
**A model with age group-by-sleep interaction**				
Change in sleep health composite	0.019	-0.003	0.040	.087
T1 Age ≤ 43 yrs	0.102	0.055	0.148	**<.001**
T1 Age ≥ 67 yrs	-0.309	-0.364	-0.253	**<.001**
Change in sleep health*T1 age ≤ 43	-0.001	-0.041	0.039	.975
Change in sleep health*T1 age ≥ 67	-0.037	-0.088	0.013	.145

Note: significance level $\alpha = .05$. Reference group in interaction models: T1 age 44 – 66 (i.e., within +/- 1 standard deviation of T1 sample median). Fully adjusted models control for sex, race/ethnicity, education, depression symptoms, body mass index, smoking status, alcohol use, and employment status. T1 = Timepoint 1; BTACT = the Brief Test of Adult Cognition by Telephone.
